# Placental IGFBP1 levels during early pregnancy and the risk of insulin resistance and gestational diabetes

**DOI:** 10.1038/s41591-024-02936-5

**Published:** 2024-04-16

**Authors:** Marie-France Hivert, Frédérique White, Catherine Allard, Kaitlyn James, Sana Majid, François Aguet, Kristin G. Ardlie, Jose C. Florez, Andrea G. Edlow, Luigi Bouchard, Pierre-Étienne Jacques, S. Ananth Karumanchi, Camille E. Powe

**Affiliations:** 1 Department of Population Medicine, Harvard Medical School, Harvard Pilgrim Health Care Institute, Boston, MA USA; 2 Diabetes Unit, Endocrine Division, Department of Medicine, Massachusetts General Hospital, Boston, MA USA; 3 Centre de Recherche du Centre Hospitalier Universitaire de Sherbrooke (CRCHUS), Sherbrooke, Quebec Canada; 4 Département de Biologie, Université de Sherbrooke, Sherbrooke, Quebec Canada; 5 Department of Obstetrics and Gynecology, Massachusetts General Hospital, Harvard Medical School, Boston, MA USA; 6 Broad Institute of MIT and Harvard, Cambridge, MA USA; 7 Center for Genomic Medicine, Massachusetts General Hospital, Boston, MA USA; 8 Department of Medicine, Harvard Medical School, Boston, MA USA; 9 Department of Biochemistry and Functional Genomics, Université de Sherbrooke, Sherbrooke, Quebec Canada; 10 Department of Medical Biology, CIUSSS of Saguenay-Lac-Saint-Jean, Saguenay, Quebec Canada; 11 Institut de Recherche sur le Cancer de l’Université de Sherbrooke (IRCUS), Sherbrooke, Quebec Canada; 12 Department of Medicine, Cedars-Sinai Medical Center, Los Angeles, CA USA

**Keywords:** Gestational diabetes, Gene expression, Predictive markers

## Abstract

Reduced insulin sensitivity (insulin resistance) is a hallmark of normal physiology in late pregnancy and also underlies gestational diabetes mellitus (GDM). We conducted transcriptomic profiling of 434 human placentas and identified a positive association between insulin-like growth factor binding protein 1 gene (*IGFBP1*) expression in the placenta and insulin sensitivity at ~26 weeks gestation. Circulating IGFBP1 protein levels rose over the course of pregnancy and declined postpartum, which, together with high gene expression levels in our placenta samples, suggests a placental or decidual source. Higher circulating IGFBP1 levels were associated with greater insulin sensitivity (lesser insulin resistance) at ~26 weeks gestation in the same cohort and in two additional pregnancy cohorts. In addition, low circulating IGFBP1 levels in early pregnancy predicted subsequent GDM diagnosis in two cohorts of pregnant women. These results implicate IGFBP1 in the glycemic physiology of pregnancy and suggest a role for placental IGFBP1 deficiency in GDM pathogenesis.

## Abstract (web summary)

Using plasma samples collected over several time points during pregnancy from three different cohorts, associations between circulating placental IGFBP1 levels, metabolic traits and birth anthropometric measurements were measured, with low IGFBP1 levels identified as a potential risk factor for gestational diabetes mellitus.

## Main

Gestational diabetes mellitus (GDM) affects one in seven pregnancies worldwide^1^ and is associated with maternal and offspring adverse health outcomes during pregnancy, at delivery and over the life course^2^. Previous research has established that a defect in insulin sensitivity (that is, excess insulin resistance) contributes to GDM^3,4^. In addition, we and others^5–10^ have shown previously that, among individuals with GDM, those with the lowest insulin sensitivity (insulin-resistant GDM) have the greatest risk of hyperglycemia-associated pregnancy complications, suggesting that reduced insulin sensitivity is a key contributor not only to GDM itself, but also to the negative health outcomes that accompany it.

The placenta is the main driver of marked changes in insulin physiology during pregnancy, including the drastic decline in insulin sensitivity, which occurs even in those without GDM. This has been attributed to hormonal factors released by the placenta that lead to insulin resistance^11^. The specific placental circulating factors that mediate the profound change in insulin sensitivity during pregnancy are still unknown, and the classically implicated pregnancy hormones (for example, human chorionic gonadotropin, human placental lactogen and placental growth hormone) have been found to correlate poorly with insulin sensitivity in pregnancy in human studies^12^. A better understanding of the placental factors driving the pregnancy-related decline in insulin sensitivity could lead to new therapeutic approaches to hyperglycemia, early identification of those at risk of developing GDM and recognition of those most likely to have GDM-related pregnancy complications. Despite the hallmark reduction in insulin sensitivity in all pregnancies, most pregnant individuals do not develop GDM. This phenomenon suggests that additional factors, as yet unknown, may contribute to the maintenance of euglycemia in pregnancy. Indeed, a variable improvement in insulin sensitivity has been reported in early pregnancy in several studies^13–15^. A systematic search for placental factors that are related to insulin sensitivity in pregnancy may also uncover those that improve it.

The overarching goal of this study was to discover new placental factors implicated in physiologic changes in insulin sensitivity during pregnancy and that contribute to GDM pathophysiology. We conducted a placental genome-wide transcriptomic study using RNA sequencing (RNA-seq) to identify genes whose expression in the placenta was associated with insulin sensitivity in pregnancy. We identified the insulin-like growth factor binding protein 1 gene (*IGFBP1*) as the most strongly associated placental transcript. IGFBP1 is a binding protein that is produced primarily by the liver outside of pregnancy and is highly expressed by the placenta^16^. IGFBP1 has been implicated in the modulation of the biological activity of insulin-like growth factor (IGF)-1 and IGF-2, which are key regulators of growth and metabolism in postnatal and fetal life^17^. Outside of pregnancy, low IGFBP1 concentrations are correlated with insulin resistance and associated with risk of incident type 2 diabetes (T2D)^18–20^ but this has not been shown in pregnancy. In the current study, using plasma samples collected from three different pregnancy cohorts with diverse backgrounds, we measured circulating IGFBP1 protein levels at several time points during and after gestation. Using these data, we investigated associations between circulating IGFBP1 levels and insulin sensitivity, other pregnancy-related metabolic traits, birth anthropometric measurements and risk of GDM.

## Results

### Participants in the placental genome-wide RNA-seq analyses

A genome-wide RNA-seq study was conducted using placental samples collected from 434 participants in the Genetic of Glucose regulation in Gestation and Growth (Gen3G) prospective pregnancy cohort^21^ (Table 1). At study entry (median, 9 weeks gestation), participants’ mean ± s.d. age was 28.7 ± 4.4 years, and median interquartile range (IQR) body mass index (BMI) was 23.8 (21.4–27.9) kg m^−2^. Individuals with diabetes present before pregnancy were excluded. Participants underwent a fasting 75 g oral glucose tolerance (75g-OGTT) in the late second trimester (median, 26 weeks gestation), during which extra plasma samples were collected to measure glucose and insulin levels at several time points to estimate insulin sensitivity (using the Matsuda index, which has been validated previously against euglycemic clamps in pregnancy^22^). At delivery (median (IQR) = 39.6 (38.7–40.3) weeks), trained research staff collected samples from the maternal-facing side of the placenta using standardized protocols for collection and storage for future analyses by RNA-seq (Methods).

**Table 1:** Characteristics of Gen3G participants included in the placental RNA-seq analyses (*n* = 434)

Characteristics	Mean (SD) or median [IQR] or *n* (%)
Maternal age (years)	28.7 (4.4)
Primigravid	154 (35.5%)
Gestational age at first trimester visit (weeks)	9.4 [8.1–11.6]
Maternal BMI at first trimester visit (kg m^−2^)	23.8 [21.4–27.9]
Gestational age at second trimester visit (weeks)	26.3 [25.9–27.3]
GDM^a^	35 (8.1%)
Insulin sensitivity, Matsuda index	6.74 [4.70–9.36]
Gestational age at delivery (weeks)	39.6 [38.7–40.3]
Fetal sex, female	202 (46.5%)

Matsuda calculated at the second trimester visit.

^a^Based on International Association of the Diabetes and Pregnancy Study Groups (IADPSG) criteria.

### Differential placental RNA expression and insulin sensitivity

**Fig. 1:**
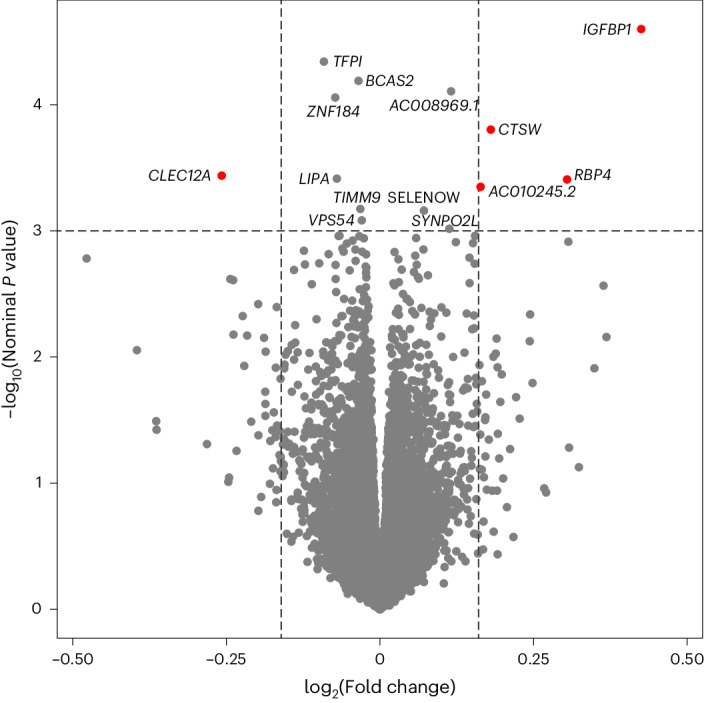
Volcano plot showing placenta RNA differential expression in relation to insulin sensitivity (Matsuda, log_2_ transformed) at 26 weeks gestation in 434 Gen3G participants. Linear model adjusted for maternal age, gravidity and maternal BMI at first trimester visit, sex of offspring, gestational age at delivery and 37 SVs (from SmartSVA package); gene names identified if *P* < 1.0 × 10^−3^. Red dots indicate genes with association *P* < 1.0 × 10^−3^ (horizontal dotted line) and absolute log_2_ fold changes greater than 5 s.d. from the mean (vertical dotted lines).

After processing and quality control (QC) of the placental RNA-seq dataset, differential expression of 15,202 genes were analyzed in relation to insulin sensitivity (Matsuda index, log_2_ transformed) in late second trimester. We identified 14 genes whose placental RNA expression levels were associated with insulin sensitivity (*P* < 1.0 × 10^−3^; Extended Data Table 1) after accounting for technical variability (37 surrogate variables (SV)), precision variables (gestational age at delivery, fetal sex) and potential confounders (gravidity, maternal age and BMI) using multivariate linear regression models. The strongest association was found between insulin sensitivity and the *IGFBP1* gene (*β* = 0.43; *P* = 2.5 × 10^−5^), where higher placental expression levels were associated with greater insulin sensitivity (Fig. 1). No strong associations were observed between the Matsuda index and other genes in IGF-related pathways, or genes encoding classic pregnancy-specific placental hormones (for example, human placental growth hormone (*GH2*), human placental lactogen (*CSH1*), prolactin (*PRL*)) or genes encoding inflammatory proteins secreted by the placenta that have been found previously to be associated with insulin sensitivity in pregnancy^12^ (Extended Data Table 2); some of the classic placental hormones (*CSH1*, *GH2*) had mappability scores <0.8, so these results should be interpreted with caution.

### Circulating IGFBP1 levels during pregnancy

Given the high levels of placental expression of *IGFBP1* (average transcript per million = 103.4) and its known secreted protein status, circulating levels of IGFBP1 (R&D systems enzyme-linked immunosorbent assay, catalog number DGB100) were measured in Gen3G participants (*n* = 837; Extended Data Table 3). Circulating levels of IGFBP1 were correlated with placental RNA expression of *IGFBP1* (Pearson *r* = 0.15; *P* = 0.002 with IGFBP1 levels at V1; *r* = 0.14; *P* = 0.005 at V2; adjusted for gestational age at each visit). Furthermore, circulating levels of IGFBP1 were measured in two additional pregnancy cohorts: the Study of Pregnancy Regulation of Insulin and Glucose (SPRING)^15^ and the Massachusetts General Hospital (MGH) Obstetrical Maternal Study (MOMS). Characteristics of participants included in SPRING and MOMS are presented in Extended Data Table 4).

**Extended Data Fig. 1:**
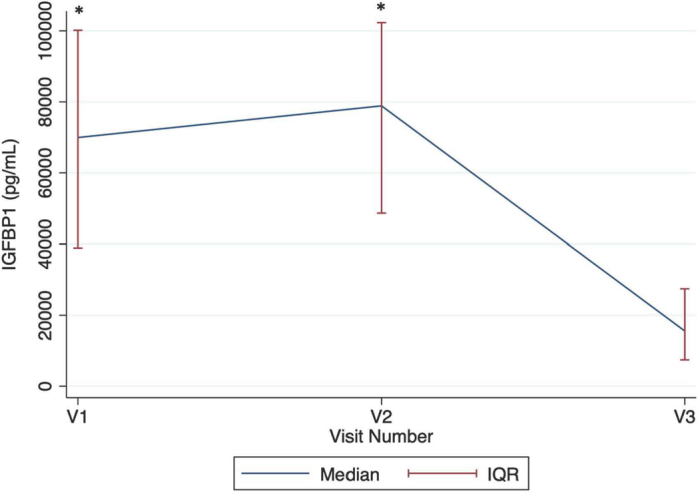
Longitudinal changes in median plasma IGFBP-1 levels across pregnancy and postpartum in normoglycemic SPRING participants (N = 65). IGFBP1 levels comparison using two-sided Wilcoxon signed-rank test, unadjusted for multiple comparisons. Exact *P* = 5.08 ×10^-15^ for differences between V3 (median 9 weeks post-partum) and V1 (median = 13 weeks gestation) and *P* = 9.18 ×10^-19^ for differences between V3 and V2 (median= 26 weeks gestation), denoted with *. Blue line is linking median value at each time point. Red bars represent interquartile range at each time point.

In SPRING participants who remained normoglycemic throughout pregnancy (*n* = 65), the median plasma levels of IGFBP1 rose between the first trimester (66,610 pg ml^−1^) and 24 to 32 weeks gestation (79,379 pg ml^−1^), then declined dramatically postpartum (16,588 pg ml^−1^; paired *t*-tests *P* < 0.001 for differences between plasma levels across pregnancy and postpartum; Extended Data Fig. 1). This pattern, combined with high placental expression levels, suggests a possible placental origin of high circulating IGFBP1 levels during pregnancy.

**Extended Data Fig. 2:**
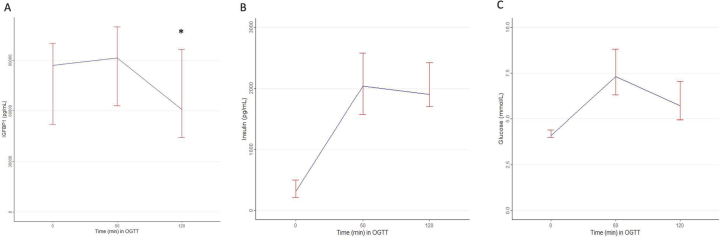
Longitudinal changes in median plasma levels of IGFBP-1, insulin, and glucose during 75g-OGTT conducted at median 26 weeks in 27 Gen3G participants. Panel A: IGFBP1 levels comparison using two-sided Wilcoxon signed-rank test: V = 125, *P* = 0.13 for differences between 60 min and fasting (0 min); V = 324, *P* = 0.0007 (denoted with *) for differences between 120 min and fasting (0 min) without adjustment for multiple comparisons. Panel B: insulin levels over three time points of OGTT. Panel C: glucose levels over three time points of OGTT. Blue lines are linking median value between time points. Red bars represent interquartile range at each time point.

In a subset of Gen3G participants (*n* = 27) in whom we assayed serial IGFBP1 levels during the 75g-OGTT (Extended Data Fig. 2), circulating IGFPB1 levels were stable over the first hour of the OGTT (median levels, fasting = 87,008 pg ml^−1^; 1 h postload = 91,485 pg ml^−1^; paired *t*-test *P* = 0.13), but declined 2 h postglucose-load (median = 60,920 pg ml^−1^; paired *t*-test *P* = 0.0007 compared with fasting). The change in plasma insulin levels from baseline to 1 h (delta insulin 0–60 min) appeared to be associated inversely with the IGFBP1 levels at 1 h (*r* = −0.39; *P* = 0.047) and at 2 h (*r* = −0.31; *P* = 0.11) during the OGTT. This is consistent with the known negative feedback regulation of *IGFBP1* expression by insulin, albeit shown previously only in hepatocytes^23^.

### Circulating IGFBP1 and insulin sensitivity in pregnancy

Higher plasma IGFBP1 levels were associated with greater insulin sensitivity in all three pregnancy cohorts examined (Table 2). The strong positive correlations (Pearson *r* = 0.5 to 0.6; *P* < 0.001) between plasma IGFBP1 levels and insulin sensitivity were consistent across different periods of pregnancy, as well as in the postpartum period (SPRING). Adjusting for maternal age and gestational age at the time of blood sampling did not influence correlations. The strength of association was attenuated modestly by further adjustment for maternal BMI, but remained highly statistically significant (*r* = 0.34–0.48, *P* < 0.001; Table 2).

**Table 2:** Cross-sectional correlations between plasma IGFBP1 levels and insulin sensitivity during and after pregnancy in the Gen3G, SPRING and MOMS cohorts

Cohort			Unadjusted correlations		Partial correlations adjusted for maternal age and gestational age at blood draw		Partial correlations adjusted for maternal age, gestational age at blood draw and maternal BMI	
	Timing	*N*	Correlation	*P* values	Correlation	*P* values	Correlation	*P* values
Gen3G	24–30 weeks gestation	816	*r* = 0.50	<0.001	*r* = 0.50	<0.001	*r* = 0.35	<0.001
SPRING	7–15 weeks gestation	156	*r* = 0.50	<0.001	*r* = 0.49	<0.001	*r* = 0.35	<0.001
	24–32 weeks gestation	119	*r* = 0.55	<0.001	*r* = 0.55	<0.001	*r* = 0.34	<0.001
	6–24 weeks postpartum	107	*r* = 0.57	<0.001	*r* = 0.58	<0.001	*r* = 0.48	<0.001
MOMS	16–20 weeks gestation	97	*r* = 0.60	<0.001	*r* = 0.59	<0.001	*r* = 0.45	<0.001

Gen3G: Pearson correlations using second trimester plasma IGFBP1 (Box–Cox transformation) and Matsuda (log transformation); maternal BMI measured at first trimester. *P* values were too small to be computed as exact *P* values.

SPRING: Pearson correlations between plasma IGFBP1 and Matsuda (log transformation) cross-sectionally at each visit; partial correlations for postpartum visit adjusted for number of weeks postpartum (instead of gestational age).

MOMS: Pearson correlations between plasma IGFBP1 and HOMA-IS (log transformation); MOMS participants include GDM cases matched to non-GDM participants (matched on gestational age and year of sample collection).

In Gen3G, correlations between plasma IGFBP1 (in the first and second trimester) and various maternal metabolic traits and neonatal anthropometric measures were assessed using Pearson correlations (Extended Data Table 5). Higher maternal BMI was associated with lower plasma IGFBP1 in the first trimester (*r* = −0.27) and in the late second trimester (*r* = −0.54; both *P* < 0.001). Plasma IGFBP1 in the late second trimester was correlated negatively with glucose (*r* = −0.28 to −0.30) and insulin levels (*r* = −0.40) during the OGTT (all *P* < 0.001). Lower IGFBP1 levels at both time points were also associated with higher birthweight *z*-scores (standardized for gestational age and sex) at delivery (*r* = −0.15 and *r* = −0.21 for IGFBP1 at the first and second trimester visits, respectively; both *P* < 0.001; Extended Data Table 5). Adjusting for maternal BMI or for maternal glucose reduced the strength of associations, but the correlations remained statistically significant (for example, second trimester IGFBP1 partial correlations with birthweight *z*-score adjusted for maternal BMI *r* = −0.12; *P* < 0.001; or adjusted for maternal glucose (glucose area under the curve (AUC) during the OGTT) *r* = −0.17; *P* < 0.001). Lower IGFBP1 at the second trimester visit was associated with higher risk of large-for-gestational (LGA) birthweight (odds ratio (OR) = 0.60 (95% confidence interval (CI) = 0.46–0.78); *P* = 0.0001); this association was reduced but remained statistically significant after adjustment for maternal BMI (OR = 0.73 (95% CI = 0.54–0.99); *P* = 0.045).

### Early pregnancy circulating IGFBP1 and GDM incidence

**Fig. 2:**
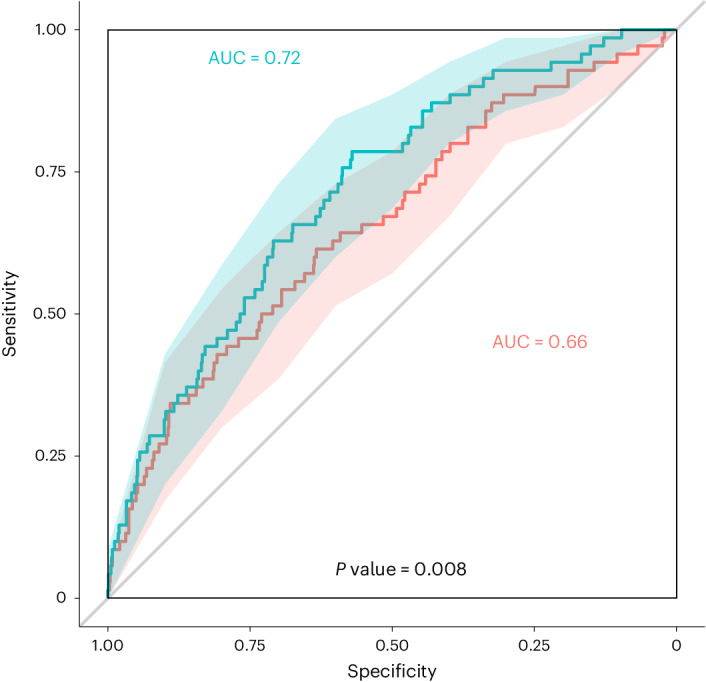
Prediction of GDM diagnosed at a median 26 weeks gestation from first trimester plasma IGFBP1 levels in 837 Gen3G participants (70 GDM cases). Red line (clinical variables only): ROC curve for GDM diagnosis based on maternal age, gravidity, family history of diabetes, gestational age at V1 and maternal BMI at V1; green line: ROC curve for GDM diagnosis based on all clinical variables plus plasma IGFBP1 levels (measured at a median of 9 weeks of gestation). GDM diagnosed by IADPSG criteria. Shaded areas: 95% CI for each curve (2,000 stratified bootstrap). Comparing AUC values with and without plasma IGFBP1 (Box–Cox transformation) using a two-sided DeLong’s test gave estimates (difference between 2 AUC) = −0.060, 95% CI = −0.104 to −0.015, *z* = −2.641, *P* = 0.008.

Prediction analyses were conducted using plasma IGFBP1 measured in early pregnancy (median 9 weeks gestation) and GDM (diagnosed with International Association of the Diabetes and Pregnancy Study Groups (IADPSG) criteria applied to a 75g-OGTT at a median of 26 weeks gestation) in Gen3G participants (*n* = 837) in addition to known clinical risk factors. Overall, 70 participants (8.4%) developed GDM (Extended Data Table 3). Early pregnancy IGFBP1 levels alone predicted risk of incident GDM with a modest receiver operating characteristic (ROC) AUC value of 0.64. A model including only clinical variables (maternal age, gravidity, family history of diabetes, maternal BMI, gestational week at blood sampling) without IGFBP1 levels yielded an ROC AUC of 0.66 (Fig. 2). A model with the same clinical variables but also incorporating early pregnancy IGFBP1 levels improved predictive ability (ROC AUC = 0.72 compared with 0.66; *P* = 0.008; Fig. 2). Using a logistic regression model, a 1 s.d. increase in plasma IGFBP1 levels in early pregnancy was associated with a greater than 50% reduction in the risk for GDM in Gen3G (OR = 0.44; IQR = 0.30–0.64; *P* < 0.001; adjusted for maternal age, gravidity, gestational age at plasma IGFBP1 measurements and maternal BMI; Table 3).

**Table 3:** First and second trimester levels of plasma IGFBP1 (per s.d. increase) and risk of GDM overall and by GDM subtype in Gen3G (OR (95% CI) from logistic regressions)

	GDM (all)	Insulin-resistant GDM	Insulin-deficient GDM
Number of GDM cases/non-GDM	*N* = 70/ 767	*N* = 34/767	*N* = 19/767
First trimester plasma IGFBP1 levels			
Model 1, unadjusted	OR = 0.597	OR = 0.488	OR = 0.676
	(0.460–0.768)	(0.336–0.697)	(0.416–1.079)
	*P* = 5.2 × 10^−5^	*P* = 6.4 × 10^−5^	*P* = 0.10
Model 2, adjusted	OR = 0.388	OR = 0.285	OR = 0.617
	(0.271–0.548)	(0.170–0.464)	(0.326–1.140)
	*P* = 3.4 × 10^−8^	*P* = 2.0 × 10^−7^	*P* = 0.12
Model 3, BMI adjusted	OR = 0.441	OR = 0.449	OR = 0.427
	(0.299–0.642)	(0.256–0.769)	(0.208–0.850)
	*P* = 1.4 × 10^−5^	*P* = 0.003	*P* = 0.02
Second trimester plasma IGFBP1 levels			
Model 1, unadjusted	OR = 0.452	OR = 0.216	OR = 0.992
	(0.345–0.586)	(0.128–0.340)	(0.626–1.605)
	*P* = 5.6 × 10^−10^	*P* = 4.9 × 10^−14^	*P* = 0.97
Model 2, adjusted	OR = 0.454	OR = 0.214	OR = 0.993
	(0.345–0.590)	(0.126–0.340)	(0.622–1.620)
	*P* = 9.2 × 10^−10^	*P* = 5.8 × 10^−14^	*P* = 0.98
Model 3, BMI adjusted	OR = 0.478	OR = 0.283	OR = 0.720
	(0.349–0.647)	(0.159–0.470)	(0.410–1.274)
	*P* = 1.1 × 10^−6^	*P* = 1.6 × 10^−7^	*P* = 0.26

Plasma IGFBP1 levels transformed using Box–Cox for optimal normal distribution, then translated in *z*-score for the logistic regression analyses. OR and 95% CI are per 1 s.d. increase of plasma IGFBP1. Exact *P* values provided, no adjustment for multiple testing.

Model 2: logistic regression models adjusted for maternal age, gravidity and gestational age at plasma samples (V1 and V2, respectively).

Model 3: logistic regression including Model 2 covariates plus maternal BMI measured at V1.

GDM diagnosis was made at a median of 26 weeks gestation.

Predictive association between early pregnancy IGFBP1 levels and GDM incidence were replicated in a nested case–control study in the MOMS cohort (*n* = 55 GDM cases, diagnosed based on Carpenter–Coustan criteria at a median of 29 weeks gestation; matched 1:2 with noncases): the OR was 0.40 (95% CI, 0.24–0.67; *P* < 0.001, adjusted for maternal age and BMI) per s.d. increase in plasma IGFBP1 (measured at a median of 17 weeks gestation). In the SPRING cohort, all GDM cases were combined (*n* = 44 total, diagnosed either in early pregnancy or at 24–32 weeks gestation based on IADPSG criteria) and predictive analyses showed an OR of 0.75 (95% CI, 0.46–1.25; *P* = 0.28; adjusted for maternal age, BMI and gestational age at blood samples) for each SD increase in plasma IGFBP1 measured in the first trimester (median, 13 weeks gestation).

### Circulating IGFBP1 in pregnancy by GDM physiologic subtype

**Fig. 3:**
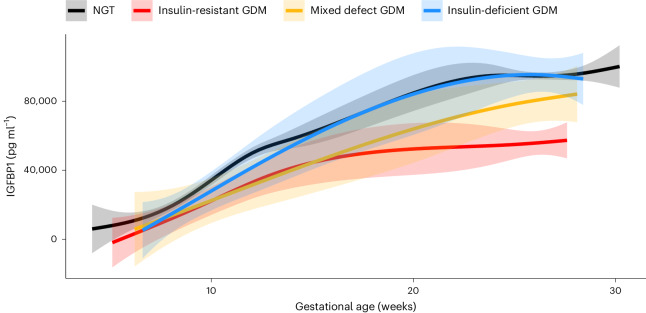
Longitudinal changes in plasma IGFBP1 levels across pregnancy in GDM subtypes and in normal glucose tolerant participants in Gen3G. Sample size in each group: NGT, *n* = 767; insulin-resistant GDM, *n* = 34; insulin-deficient GDM, *n* = 19; and mixed defect GDM, *n* = 12. Lines represent the levels of circulating IGFBP1 (not transformed) from the first trimester visit to the late second trimester visit at exact gestational age of the measure, smoothed by a generalized additive model with parameter estimation via restricted maximum likelihood using ‘stat_smooth’ function from the ggplot2 package. Shaded areas represent 95% CIs.

Given the strong association between plasma IGFBP1 and insulin sensitivity in pregnancy, the longitudinal changes in plasma IGFBP1 across pregnancy in different physiologic subtypes of GDM (as defined previously^10^) and in participants with normal glucose tolerance (NGT) were investigated in Gen3G (Fig. 3). All GDM subtypes had lower mean plasma IGFBP1 levels in early pregnancy compared with the NGT group. However, the insulin-resistant GDM group had a blunted increase in IGFBP1 levels between the first and second trimester; in contrast, in those with insulin-deficient GDM, IGFBP1 levels reached similar levels to those in the NGT group during the second trimester (Fig. 3). The group who had GDM with both insulin resistance and insulin deficiency (mixed defect GDM) showed an IGFBP1 trajectory that was intermediate between the other GDM subtypes.

In Gen3G, low IGFBP1 levels in first trimester were associated with subsequent diagnosis of both insulin-resistant GDM and insulin-deficient GDM with ORs ~0.4 (in fully adjusted models, including maternal BMI) similar to prediction models where the outcome was all GDM (Model 3; Table 3). However, IGFBP1 levels in the second trimester were associated only with insulin-resistant GDM (OR = 0.28 (0.16–0.47) per s.d. increase in IGFBP1 levels; *P* < 0.001); there was no statistically significant association between second trimester IGFBP1 plasma levels and insulin-deficient GDM (Table 3).

## Discussion

In this study, using genome-wide RNA-seq of placental tissue, we identify *IGFBP1* as a key placental transcript associated with insulin sensitivity in human pregnancy. Our findings implicate IGFBP1 deficiency in GDM pathophysiology. We show that circulating IGFBP1 levels rise during pregnancy and are much higher in pregnancy than in the nonpregnant state, supporting the contribution of placental and/or decidual *IGFBP1* to elevated circulating IGFBP1 in pregnancy. In three independent pregnancy cohorts, we demonstrate a strong and consistent correlation between higher circulating IGFBP1 and greater insulin sensitivity (lesser insulin resistance), uncovering a potential compensatory mechanism in euglycemic pregnancy. Moreover, we show that low plasma IGFBP1 levels in the first trimester of pregnancy predict the later diagnosis of GDM, independent of maternal clinical risk factors (including BMI). Finally, we note that the normal pregnancy rise in IGFBP1 levels is attenuated in insulin-resistant GDM, suggesting that a defect in placental IGFBP1 release may contribute specifically to this GDM physiologic subtype.

In placental tissues, *IGFBP1* expression has been detected previously in decidual cells and in fetal placental macrophages or Hofbauer cells^24^; however, there is limited knowledge of *IGFBP1* regulation and actions in pregnancy. Given that our samples were collected from the maternal-facing side, it is possible that *IGFBP1* expression and release from decidual cells contributed to IGFBP1 circulating levels. In vitro experiments using decidualized human endometrial stromal cells have shown *IGFBP1* regulation by cAMP, progesterone and relaxin^25,26^—the latter two being critical hormones for the establishment and maintenance of pregnancy^27^. Outside of pregnancy, *IGFBP1* is expressed almost exclusively by the liver^16^ and its production is regulated by insulin, which inhibits its gene transcription in hepatocytes^28^. This is consistent with low IGFBP1 levels reflecting insulin resistance and hepatic steatosis in nonpregnant adults and children^18,19,29^. Our observations that plasma IGFBP1 levels decline after a plasma insulin rise in response to an oral glucose load introduce the possibility that insulin may downregulate the production and/or release of IGFBP1 from the placenta/decidua, similar to the downregulation observed in hepatocytes^28^. It is also possible that other insulin-sensitive endocrine factors, such as adiponectin, regulate *IGFBP1* expression in placental cells^30^.

Functional studies suggest that IGFBP1 binds IGF-1 and IGF-2 with equal affinity and can either inhibit or enhance IGF actions, depending on the context^23^. In postnatal life, IGF-1 is the main active growth factor and is essential for normal growth during childhood and adolescence, whereas, during fetal development, both IGF-1 and IGF-2 are key regulators of fetal growth^23,31^. Outside of pregnancy, IGF-1 enhances insulin sensitivity by suppressing hepatic glucose production^32,33^ and promoting glucose uptake in peripheral tissues^34,35^. *IGF2* is a highly expressed placental imprinted gene that is a key regulator of fetal growth in mammals^36^. In a recent study, pregnant mice with an *IGF2* deletion specific to placental endocrine cells did not develop the normal insulin resistance of pregnancy and gave birth to fetuses that were growth-restricted and hypoglycemic^37^. In general, IGFs have higher affinity for IGFBPs than for cellular IGF-receptors and, thus, IGFBPs, sometimes in combination with acid-labile subunits, often act as inhibitors of IGF biological activity^25^. PAPPA and PAPPA2 are two key proteinases released by the placenta that allow the cleavage of IGFBPs from IGFs; however, these proteinases do not cleave IGFBP1 (refs. ^38,39^). IGFBPs may also function as a circulating pool of IGFs by prolonging their half-lives and creating IGF reservoirs^17,23^. In addition, IGFBP1 has putative IGF-independent effects, and may activate PI3K/AKT signaling pathways involved in postreceptor insulin signaling directly^40^. In line with this, in vivo injection of an active IGFBP1 peptide improved insulin sensitivity in a diet-induced obesity mouse model^41^. These diverse mechanisms of action might explain some of the inconsistencies from previous animal studies attempting to establish the effects of IGFBP1 on glucose regulation^42–44^. Future studies of gestational glycemic pathophysiology should investigate the interrelations of IGFs with the different IGFBPs and their regulation from acid-labile subunits and PAPPAs in the context of pregnancy.

None of these previous studies provide insights into the specific role that IGFBP1 may have in pregnancy, when there are high circulating levels of IGFs, which are suspected to influence glucose metabolism^23,37^. We speculate that placental/decidual release of IGFBP1 may regulate insulin sensitivity in pregnancy—via direct and/or indirect effects—contributing physiologically to homeostatic mechanisms to balance maternal and fetal nutrient needs. An alternative explanation is that low levels of IGFBP1 in GDM are a consequence of hyperinsulinemia with another upstream cause, but this would not be consistent with the rise of circulating IGFBP1 throughout pregnancy (which is characterized by progressive hyperinsulinemia). In the context of GDM pathophysiology, based on our findings, in individuals with insulin-resistant GDM, we speculate that the placenta may be unable to produce increasing amounts of IGFBP1 as pregnancy progresses; this deficiency in circulating IGFBP1 could contribute to excessive insulin resistance, and thus to maternal hyperglycemia detected in the late second trimester in this GDM subtype. In individuals with insulin-deficient GDM, IGFBP1 amounts were low in the first trimester but amounts during the second semester were on par with those without GDM, suggesting that other pathophysiologic factors contribute to hyperglycemia in this GDM subtype. Given the differences in IGFBP1 in different GDM subtypes, and increasing recognition in the field that GDM is a heterogeneous condition^45^, our finding of persistently lower IGFBP1 levels in the second trimester of pregnancies affected by insulin-resistant GDM may have implications for GDM precision medicine^46,47^. Our findings suggest that, in cases of insulin-resistant GDM, the placenta does not increase IGFBP1 production sufficiently; if this association is demonstrated to be causal, this opens the door to a new therapeutic target for this GDM subtype. Beyond GDM, the association between lower circulating IGFBP1 levels and higher birthweight is in line with similar observations in an earlier report^48^ and suggests a potential explanation for the greater risk of LGA birthweight that we observed previously in instances of insulin-resistant GDM^10^.

Accurately predicting GDM diagnosis in later pregnancy based on early pregnancy markers could allow development and implementation of interventions—such as counseling on diet and exercise—to prevent GDM and its complications. However, most predictive models that rely on established clinical risk factors perform poorly^49,50^ and, thus, there has been a search for reliable and replicable biomarkers. We found that low levels of circulating IGFBP1 in early pregnancy predict later diagnosis of GDM in a large population-based cohort (Gen3G), with external replication and consistent effect sizes in a separate cohort (MOMS). The effect size was more modest and not statistically significant in a cohort study of participants who all had GDM risk factors (SPRING); these inclusion criteria may have diminished the predictive ability of circulating IGFBP1 in this population. Previous studies have been inconsistent with regard to circulating IGFBP1 as a predictive biomarker for GDM, with only one study reporting on IGFPB1 levels measured before 20 weeks of gestation^51^. Our ROC analyses showed that circulating IGFBP1 levels in early pregnancy have a predictive ability beyond that of established GDM risk factors (including maternal BMI and family history of diabetes); however, the moderate ROC AUC value in a model that included IGFBP1 levels along with these clinical factors suggests that additional biomarkers would be necessary for clinical utility. Future studies could also investigate whether urinary levels of IGFBP1 in pregnancy can predict GDM or characterize subtypes, which would be convenient for patients and clinicians. We do not know whether women who developed GDM in our study had low IGFBP1 levels before pregnancy, thus pre-pregnancy assessment of IGFBP1 should be considered in future studies of pre-conception interventions aiming to optimize metabolic outcomes in pregnancy.

Our investigation has several strengths. We included a large number of placental samples in our expression profiling, used transcriptome-wide RNA-seq and leveraged an agnostic approach to implicate genes and their products in insulin sensitivity during pregnancy. Furthermore, we examined not only placental expression of *IGFBP1*, but also circulating IGFBP1 levels in three pregnancy cohorts. Our analyses included measurement of circulating IGFBP1 levels over a longitudinal timeframe that spanned both pregnancy and postpartum. In addition, we used an OGTT-based measure of insulin sensitivity that has been validated against euglycemic clamps in pregnancy. Our study also had some limitations. Although we had a large overall sample size, the number of GDM cases was somewhat modest, and the sample size for each GDM physiologic subtype was even more limited. We conducted RNA-seq on bulk samples, including placental and decidual cells, thus we cannot confirm the exact cellular source of IGFBP1. Although we were able to tie placental/decidual RNA expression and circulating IGFBP1 levels to detailed physiologic phenotyping, our study was observational and thus cannot establish mechanisms or causality for the associations we observed.

In conclusions, starting from agnostic and unbiased placental gene expression profiling, we implicated IGFBP1 in insulin sensitivity during pregnancy. *IGFBP1* was expressed highly in our placental samples and maternal IGFBP1 levels are elevated markedly during gestation, increasing across pregnancy and dropping substantially postpartum. Both placental and circulating IGFBP1 levels are correlated strongly and consistently with maternal insulin sensitivity. A deficiency of circulating IGFBP1 in early pregnancy predicts the diagnosis of GDM in the late second trimester, independent of clinical GDM risk factors in two different pregnancy cohorts. We demonstrated distinct IGFBP1 trajectories in different physiologic subtypes of GDM, with insulin-resistant GDM lacking the expected increase in circulating IGFBP1 across gestation. Future studies should address whether IGFBP1 has direct or indirect effects on tissues that regulate maternal insulin sensitivity during pregnancy. If IGFBP1 is causally implicated in gestational glycemic regulation, new therapeutic approaches based on IGFBP1 replacement as an insulin sensitizer could be envisioned and tested for precision prevention or treatment of GDM.

## Methods

All three human cohorts included in this study recruited pregnant individuals who are all female (sex as a biological determinant) given that only biological female can experience pregnancy. We acknowledge that not all pregnant individuals self-identify as women (gender). Our study aimed at understanding biology of glucose regulation in pregnancy, thus our analyses apply to female individuals. All participants provided informed consent.

### Gen3G cohort

#### Population

Gen3G is a prospective population-based cohort that recruited pregnant women from 1 January 2010 to 30 June 2013 at the Centre Hospitalier Universitaire de Sherbrooke (CHUS), located in the province of Quebec (Canada). Participants were demographically representative of the greater population of the region^21^. Each study participant provided informed written consent, and the study protocols were reviewed by the ethical committees from CHUS, and from the Harvard Pilgrim Health Care Institute.

We recruited 1,024 pregnant women without preexisting diabetes in the first trimester (diabetes diagnosis from self-report or biochemical screening with HbA1c ≥6.5%). Exclusion criteria for enrollment in the cohort were nonsingleton pregnancies or regular use of medications that influence glucose regulation. We collected measurements and blood samples from mothers at a first trimester visit (V1) conducted between 5 and 16 weeks of gestation (median 9 weeks), and in the late second trimester (V2) at 24 to 30 weeks of gestation (median 26 weeks; the time of universal GDM screening). We collected placental samples in addition to data on mothers and offspring at delivery.

#### Variables collection and measurements

At V1, we collected demographic data and previous medical and obstetric history; we performed standardized anthropometric measurements. Trained research staff measured weight with a calibrated scale and height with a standardized stadiometer. We calculated first trimester BMI as weight divided by squared height (kg m^−2^). At V1, we also collected additional blood samples that were drawn during the 50 g glucose challenge test (GCT, performed in 95% of participants). For the current study, we excluded participants who had a first trimester random glucose or 1 h-glucose post-GCT >10.3 mmol l^−1^ (overt hyperglycemia per national guidelines at the time) as we were interested in GDM incidence (ascertained with universal testing at 24–30 weeks).

At V2, we performed similar anthropometric measurements and questionnaires as at V1. V2 occurred at the time of the fasting 75g-OGTT, which was standard clinical practice for screening and diagnosis of GDM at CHUS. We collected additional blood samples at the fasting, 1 h and 2 h time points of the 75g-OGTT to measure insulin at each time point in addition to glucose. We measured glucose levels via the hexokinase method (Roche Diagnostics; CHUS biochemistry laboratory) as soon as samples were collected. We measured insulin levels via multiplexed particle-based flow cytometric assays (Human Milliplex MAP kits; EMD Millipore) from the previously frozen plasma samples (stored at −80°C until measurement). We estimated insulin sensitivity using the Matsuda Index^52^ (using glucose and insulin values during the OGTT), as previously validated against euglycemic clamps performed in pregnancy^22^.

At delivery, we collected newborn age and sex at birth using medical records, in addition to details from the end of pregnancy and delivery complications. Trained study staff collected placentas within 30 min of delivery using a standardized protocol. In brief, a 1 cm^3^ placental tissue sample was collected from the maternal-facing side, including decidual tissue (within a 5 cm radius of the corresponding location of cord insertion on the other side). Each collected sample was immediately put in RNA-Later for at least 24 h at 4 °C before storage at −80 °C until RNA extraction.

#### RNA extraction, sequencing and QC

We extracted total RNA (average, 19.7 ± 7.1 µg) and checked the quality of each sample using an Agilent Bioanalyzer to determine the RNA integrity number (average RNA integrity number = 6.7 ± 0.8). We shipped samples (3 µg) with an RNA integrity number value ≥5 to the Broad Institute for sequencing. In a second round of sample QC at the Broad Institute (Caliper Life Sciences LabChip GX system), the RNA quality score for each sample ranged from 3.3 to 7.8 (average RNA quality score = 5.9). We submitted all samples with an RNA quality score value of 4 or higher for RNA sequencing (*n* = 466). We completed library preparation with 250 ng of each sample, using an automated variant of the Illumina TruSeq Stranded mRNA Sample Preparation Kit (Illumina, catalog number RS-122-2103). We performed Flowcell cluster amplification and sequencing according to the manufacturer’s protocols using the Illumina HiSeq 4000, to generate 101-bp paired-end reads, average of 113 million total reads (range 33 million to 378 million) per sample.

In line with best practice and the GTEx v.8 pipeline^53^, we applied STAR v.2.5.3a^54^ to align FASTQ/FASTA files to the human GRCh38 reference genome, using the parameters specified at https://github.com/broadinstitute/gtex-pipeline. Duplicate reads were marked using Picard MarkDuplicates, and expression was quantified with RNASeQC v.2.3.6 using the GENCODE v.26 annotation^55^.

Following quantification, we applied additional QC steps. Of the 466 samples sequenced, we excluded those with >1% of outlier genes (>3 times the IQR above Q3 or >3 IQR below Q1), leaving 459 samples for our final analytical dataset. Among these, we had complete data on the phenotype of interest (Matsuda index) and covariates for 434 samples. Before differential gene expression analysis, we removed genes with low abundance, keeping only those genes with at least a count of six reads and a transcript per million values >0.5 in a minimum of 20% of samples, as well as average mappability ≥0.8. After QC, 15,202 genes remained. Before differential expression analysis, we performed between-sample normalization using the R statistical software package edgeR^56^, then normalized and transformed gene counts to log_2_ counts per million reads using Voom from the Limma R package^57^. The Gen3G placental RNA-seq data are available on dbGAP (https://www.ncbi.nlm.nih.gov/projects/gap/cgi-bin/study.cgi?study_id=phs003151.v1.p1).

### Study of pregnancy regulation of insulin and glucose

SPRING is a longitudinal cohort study of pregnant participants with risk factors for diabetes that was conducted in 2015–2021. Participants were eligible if they were at <15 weeks gestation and had a history of GDM, family history of diabetes or GDM, or if they had BMI ≥ 25 kg m^−2^ and had one additional risk factor according to American Diabetes Association guidelines^58^. Participants gave informed consent and underwent a fasting 75g-OGTT at <15 weeks gestation, 24–28 weeks gestation and 6–12 weeks postpartum. The latter two study visit windows were widened to 24–32 weeks gestation and 6–24 weeks postpartum to promote participant retention (including during the COVID-19 pandemic). We measured glucose and insulin levels as previous described^15^. The Matsuda index was calculated from the glucose and insulin levels measured during the OGTT^15^. GDM was diagnosed according to IADPSG criteria applied to the OGTT at the pregnancy study visits. Most participants that met IADPSG criteria at the first visit were not retested at the second visit. Blood samples from each study visit were collected in EDTA plasma tubes, processed and frozen at −80 °C for future analysis. The study was approved by the Mass General Brigham Institutional Review Board.

### MGH obstetrical maternal study

MOMS was conducted from 1998 to 2006 (ref. ^59^). Participants were eligible if they were receiving prenatal care at MGH. Participants provided written informed consent and were enrolled at their first prenatal visit where they donated an extra blood sample from a clinical blood draw. A subset of participants in 2001–2006 volunteered to return to donate fasting blood and urine samples at 16–20 weeks gestation. Glucose and insulin levels were measured as previously described^60^. Fasting plasma samples were frozen at −80 °C and stored for future analyses. At 24–28 weeks gestation, participants without preexisting diabetes underwent universal screening for GDM with a nonfasting 50 g GCT. If the venous blood glucose 1 h after the GCT was ≥140 mg dl^−1^, patients were referred for a diagnostic 3-h 100g-OGTT. For this analysis, we included individuals whose OGTT results met Carpenter–Coustan criteria for GDM (≥2 abnormal values). Of these participants with GDM, 55 had remaining fasting samples available for analysis. We matched control participants with normal GCT results (two for each GDM case) on year of sample collection and gestational age at sample collection. We preferentially selected control samples on which fasting glucose and insulin had previously been measured on the sample collected at 16–20 weeks gestation. We calculated HOMA-2S from fasting glucose and insulin values to estimate insulin sensitivity^61^ (https://www.rdm.ox.ac.uk/about/our-clinical-facilities-and-units/DTU/software/homa).

Institutional review board approval was obtained for participants from each of the cohorts (Gen3G, SPRING, MOMS) following the principles outlined in the Declaration of Helsinki. All enrolled participants provided written informed consent before study procedures.

### Bioassays for circulating IGFBP1

We measured circulating IGFBP1 in plasma samples from all three cohorts in the same laboratory using a commercially available enzyme-linked immunosorbent assay that measures free IGFBP1 (R&D systems, catalog number DGB100). The precision for the assays were: intra-assay coefficients of variation of 5.6% and inter-assay coefficients of variation of 9.5%. We measured IGFBP1 levels in a blinded fashion, and we followed protocol for measurement per manufacturer’s instructions.

### Statistical analyses

For characteristics of participants in all three cohorts, we reported normally distributed continuous variables as mean ± s.d., non-normally distributed continuous variables as median and IQR, and categorical variables as percentages. We used a log_2_ transformation for Matsuda index (to approach a normal distribution) in the differential placental RNA expression analyses.

#### Placental differential expression analyses using RNA-seq data in Gen3G

We adjusted models for maternal age, gravidity, maternal BMI at the first trimester visit, sex of offspring and gestational age at delivery, in addition to computed SVs to account for unmeasured sources of variability, including batch effects and cell types. We used the EstDimRMT function from the R package isva^62^ to estimate the number of SVs to include given the residuals from the regression of Matsuda and biological covariates from the normalized counts, which resulted in 37 SVs computed by the R package SmartSVA^63^ recommended for our processed RNA-seq dataset. We used Limma^64^ to identify differentially expressed genes with log_2_ Matsuda as a continuous independent variable. We reported genes that had differential expression in relation to Matsuda with *P* < 1.0 × 10^−3^.

#### Circulating IGFBP1 correlation analyses

We carefully assessed plasma IGFBP1 distribution and, after considering different potential transformations, we used a Box–Cox transformation for plasma IGFBP1 levels in Gen3G (from MASS package^65^ in R) since it was the best way to approximate a normal distribution. We conducted analyses in SPRING and MOMS cohorts using plasma IGFBP1 levels without transformation, given distributions that were relatively normal. We used Pearson correlations between circulating IGFBP1 levels and Matsuda index (log transformed) in all three cohorts; we used partial correlations to assess the associations while taking into account maternal age, gestational age at blood draw and maternal BMI. In Gen3G, we also used Pearson correlations to assess associations between plasma IGFBP1 (Box–Cox transformed) and maternal metabolic markers, as well as newborn anthropometry (transformed if needed).

#### Circulating IGFBP1 and risk of GDM analyses

We conducted logistic regression analyses with the levels of circulating IGFBP1 as the independent variable and GDM as the dependent variable in Gen3G and SPRING; in MOMS, due to the matched case–control design of GDM cases to controls, we used conditional logistic regression. In Gen3G and SPRING, we used international criteria (IADPSG)^66^ to ascertain GDM, whereas in MOMS we used the Carpenter–Coustan criteria^67^. In Gen3G, we additionally subclassified GDM by the insulin physiology defect driving hyperglycemia (insulin-resistant GDM, insulin-deficient GDM or mixed defect GDM, as previously described^10^). We first built unadjusted logistic regression analyses (Model 1). We adjusted for maternal characteristics (maternal age, gravidity, gestational age at plasma samples) in Model 2 and additionally adjusted for maternal BMI in Model 3. We calculated profiled log-likelihood CIs along with likelihood ratio test *P* values (using MASS^65^ and glmglrt (https://CRAN.R-project.org/package=glmglrt) packages^47^ in R). In SPRING and MOMS cohorts, we employed similar modeling strategies using maximum likelihood dichotomous logistic models.

We conducted GDM predictive analyses using ROC curves in Gen3G to compare the predictive ability of first trimester (V1) plasma IGFBP1 levels in addition to commonly measured GDM clinical risk factors (maternal age, gravidity, family history of diabetes, gestational age at V1 and maternal BMI at V1). We compared the ROC AUC values using all the clinical factors with and without first trimester (V1) plasma IGFBP1 levels (after Box–Cox transformation). We compared the ROC AUC values from nested models using DeLong’s test using the roc.test function from the pROC package in R^68^. We considered differences between AUC values to be statistically significant if *P* < 0.05. In Gen3G, we performed analyses using R v.4.3.0 (https://www.R-project.org), STATA and SPSS v.28 only for partial correlations. In SPRING and MOMS, we performed analyses using Stata/IC v.16.1. Original code developed for placenta RNA-seq differential expression analysis in Gen3G available at https://github.com/labjacquespe/diff-exp.

### Reporting summary

Further information on research design is available in the Nature Portfolio Reporting Summary linked to this article.

## Online content

Any methods, additional references, Nature Portfolio reporting summaries, source data, extended data, supplementary information, acknowledgements, peer review information; details of author contributions and competing interests; and statements of data and code availability are available at 10.1038/s41591-024-02936-5.

### Supplementary information

Reporting Summary

## Figures and Tables

**Extended Data Table 1:**
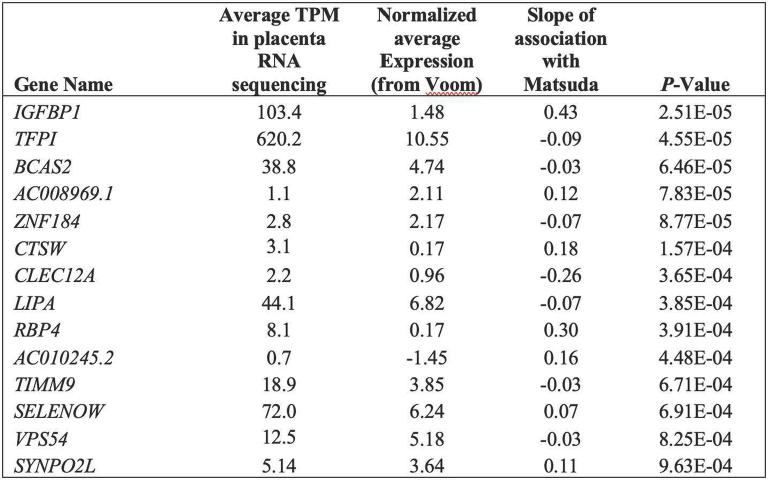
Placenta RNA differential expression in relation to Matsuda (log2 transformed) in second trimester of pregnancy in n = 434 participants of Gen3G (*P*-values < 1.0×10^-3^)

Linear model adjusted for maternal age, gravidity, maternal BMI at first trimester visit, sex of offspring, and gestational age at delivery, and 37 SVs (obtained from *SmartSVA* package). TPM: Transcript Per Million.

**Extended Data Table 2:**
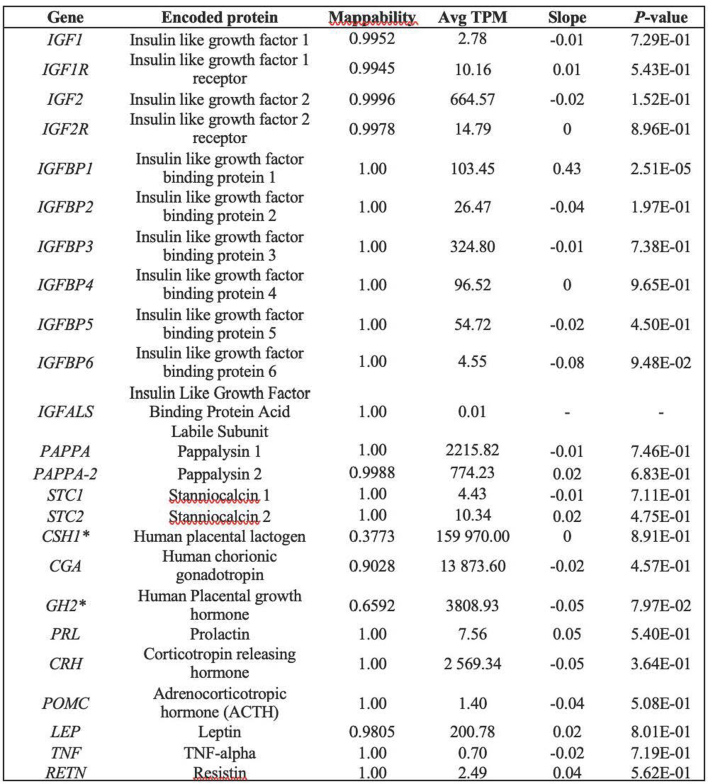
Look-ups of IGF pathways related genes, classic pregnancy hormones, and inflammatory proteins in Gen3G placental RNA sequencing dataset (n = 434) and associations with Matsuda index (log2 transformed)

Linear model adjusted for maternal age. gravidity. maternal BMI at first trimester visit. sex of offspring. and gestational age at delivery. and 37 SVs (obtained from *SmartSVA* package)

* Initially excluded from the main RNA genome-wide analysis because of an average gene mappability score < 0.8, *CSH1* and *GH2* were included in this table based on their importance for pregnancy related biology.

**Extended Data Table 3:**
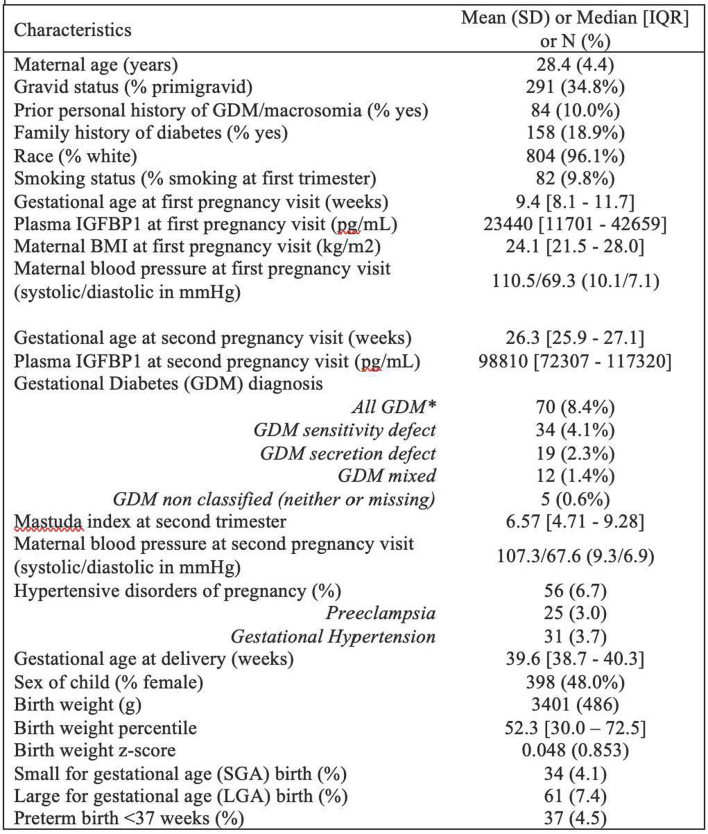
Characteristics of 837 Gen3G participants contributing to plasma IGFBP1 analyses

Missing data limiting N of following variables: Maternal blood pressure 1^st^ trimester, smoking status, family history of diabetes and IGFBP-1 at second pregnancy visit n = 836. Maternal blood pressure 2^nd^ trimester n = 835. Matsuda index n = 817; gestational age at delivery, preterm birth and child sex n = 829. Birthweight n = 828. Birthweight percentile, z-score, SGA and LGA n = 826. IGFBP1 was measured at second visit using fasting plasma samples; plasma at first visit collected non-fasting (including N = 791 samples collected 1 h post 50g-glucose challenge). * GDM diagnosed by International Association of the Diabetes in Pregnancy Study Groups (IADPSG) criteria. Hypertensive disorders of pregnancy include pre-eclampsia and gestational hypertension.

**Extended Data Table 4:**
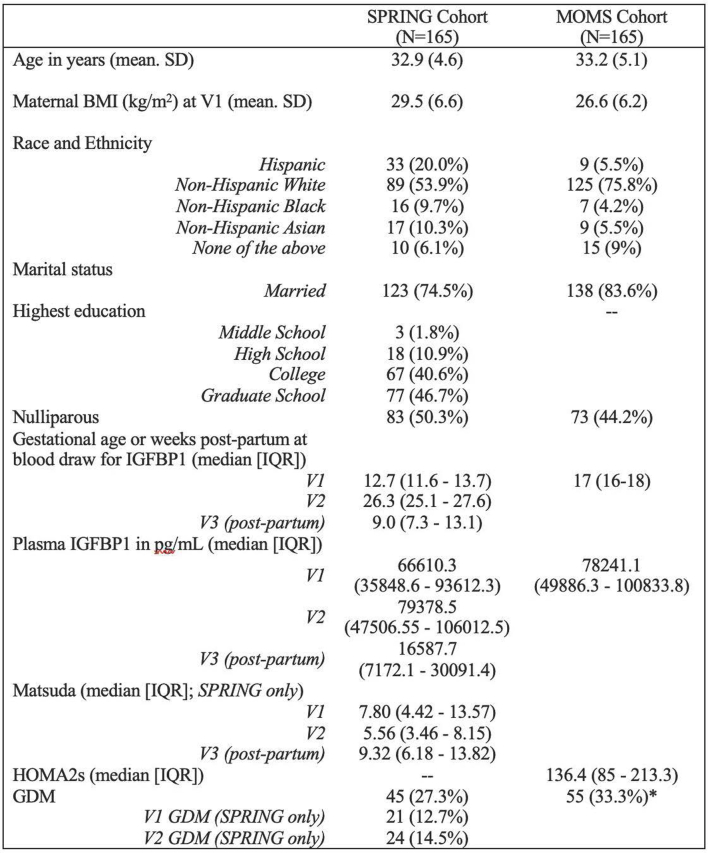
Characteristics of participants in SPRING and MOMS cohorts

*In the MOMS cohort. 55 cases of GDM were selected and matched 2:1 on sample collection year and gestational age with controls who did not have GDM; GDM was diagnosed based on Carpenter-Coustan criteria at median of 29 weeks of gestation. SPRING participants were all screened with 75g-OGTT at both first and second trimester visits; GDM was diagnosed using IADPSG criteria.

**Extended Data Table 5:**
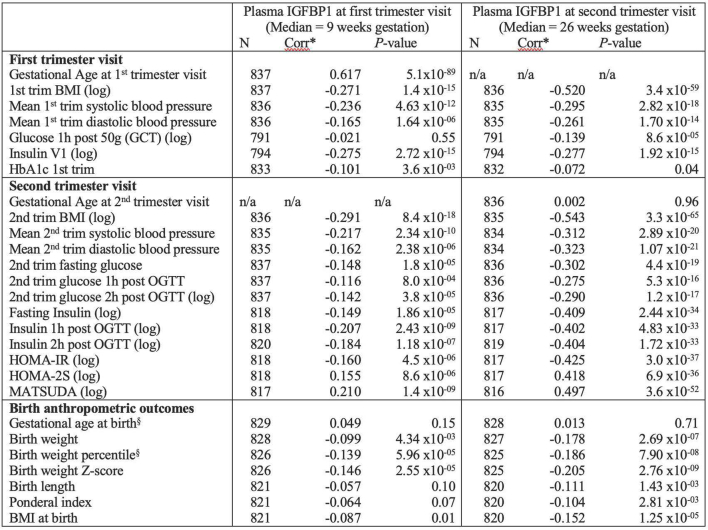
Correlations of plasma IGFBP1 levels (at the first and second trimester visits) with pregnancy metabolic traits and birth anthropometry in Gen3G

*Pearson Correlation using IGFPB1 levels (Box–Cox transformation); other variables were transformed using log when appropriate to approximate normal distribution ^§^Spearman correlation because non-normal even with log transformation. All tests are two-sided.

## Data Availability

The Gen3G placental RNA-seq data and pregnancy phenotypes are available on dbGAP (https://www.ncbi.nlm.nih.gov/projects/gap/cgi-bin/study.cgi?study_id=phs003151.v1.p1).

## Code availability

Original code developed for placenta RNA-seq differential expression analysis in Gen3G is available at https://github.com/labjacquespe/diff-exp.
