# Supplementary material for: Placental IGFBP1 levels during early pregnancy and the risk of insulin resistance and gestational diabetes
Source: Nat Med. 2024 Apr 16;30(6):1689–95. doi: 10.1038/s41591-024-02936-5 (PMC11186792; doi:10.1038/s41591-024-02936-5)
Supplement: Supplementary file 1 — Reporting Summary [file 41591_2024_2936_MOESM1_ESM.pdf]

nature portfolio

Corresponding author(s): Marie-France Hivert

Last updated by author(s): Mar 12, 2024

Reporting Summary

Nature Portfolio wishes to improve the reproducibility of the work that we publish. This form provides structure for consistency and transparency in reporting. For further information on Nature Portfolio policies, see our Editorial Policies and the Editorial Policy Checklist.

Statistics

For all statistical analyses, confirm that the following items are present in the figure legend, table legend, main text, or Methods section.

n/a	Confirmed	
☐	☒	The exact sample size (*n*) for each experimental group/condition, given as a discrete number and unit of measurement
☐	☒	A statement on whether measurements were taken from distinct samples or whether the same sample was measured repeatedly
☐	☒	The statistical test(s) used AND whether they are one- or two-sided<br>*Only common tests should be described solely by name; describe more complex techniques in the Methods section.*
☐	☒	A description of all covariates tested
☐	☒	A description of any assumptions or corrections, such as tests of normality and adjustment for multiple comparisons
☐	☒	A full description of the statistical parameters including central tendency (e.g. means) or other basic estimates (e.g. regression coefficient) AND variation (e.g. standard deviation) or associated estimates of uncertainty (e.g. confidence intervals)
☐	☒	For null hypothesis testing, the test statistic (e.g. *F*, *t*, *r*) with confidence intervals, effect sizes, degrees of freedom and *P* value noted<br>*Give P values as exact values whenever suitable.*
☒	☐	For Bayesian analysis, information on the choice of priors and Markov chain Monte Carlo settings
☒	☐	For hierarchical and complex designs, identification of the appropriate level for tests and full reporting of outcomes
☐	☒	Estimates of effect sizes (e.g. Cohen's *d*, Pearson's *r*), indicating how they were calculated

Our web collection on statistics for biologists contains articles on many of the points above.

Software and code

Policy information about availability of computer code

Data collection	no specific software was used for data collection
Data analysis	Github link for differential expression analysis - placenta RNA sequencing in Gen3G<br>https://github.com/labjacquespe/diff-exp

For manuscripts utilizing custom algorithms or software that are central to the research but not yet described in published literature, software must be made available to editors and reviewers. We strongly encourage code deposition in a community repository (e.g. GitHub). See the Nature Portfolio guidelines for submitting code & software for further information.

Data

Policy information about availability of data

All manuscripts must include a data availability statement. This statement should provide the following information, where applicable:

- Accession codes, unique identifiers, or web links for publicly available datasets
- A description of any restrictions on data availability
- For clinical datasets or third party data, please ensure that the statement adheres to our policy

The Gen3G placental RNA-seq data is available on dbGAP https://www.ncbi.nlm.nih.gov/projects/gap/cgi-bin/study.cgi?study_id=phs003151.v1.p1

Research involving human participants, their data, or biological material

Policy information about studies with human participants or human data. See also policy information about sex, gender (identity/presentation), and sexual orientation and race, ethnicity and racism.

Reporting on sex and gender	Our study included pregnant individuals who were assigned female at birth. Participants also generally identified as cis-gender women. At delivery, we collected newborn sex at birth using medical records, which we used as a biologic covariate given sex differences in placental biology.
Reporting on race, ethnicity, or other socially relevant groupings	We reported race/ethnicity composition of each cohort included in this report (Gen3G, SPRING, MOMS - see Supplementary Tables 3 and 4). We consider race/ethnicity as a social construct. Our investigations are focused on biology and physiology, thus we did not include race as a social construct into our covariates.
Population characteristics	Gen3G is a prospective population-based cohort which recruited pregnant women from January 2010 to June 2013 at the Centre Hospitalier Universitaire de Sherbrooke (CHUS), located in the province of Quebec (Canada). Participants were demographically representative of the greater population of the region. Mean age was 28 years old, about one third was primigravid, 96% self-identified as white.<br>The Study of Pregnancy Regulation of INsulin and Glucose (SPRING) is a longitudinal cohort study of pregnant participants with risk factors for diabetes that was conducted in 2015-2021. Mean age was 33 years old, about half were nulliparous, and 20% self-identified as Hispanic/Latinx, 10% as Black, 10% as Asian, and 54% as white.<br>The MGH Obstetrical Maternal Study (MOMS) was conducted from 1998-2006. Race-ethnicity was reported as recorded in the electronic medical record. While this was generally self-reported, we do not have verification of this. Mean age was 33 years old, 44% were nulliparous, 6% were Hispanic, 4% were Black, 6% were Asian, and 76% as white.
Recruitment	Gen3G: During the recruitment period, all pregnant women presenting for their first trimester blood sampling were presented the information about the study. Exclusion criteria for enrollment in the cohort were non-singleton pregnancies or regular use of medications that influence glucose regulation, or preexisting diabetes in the first trimester (diabetes diagnosis from self-report or biochemical screening with HbA1c ≥6.5%)<br>SPRING: Women receiving care in the Obstetric Department at Massachusetts General Hospital (Boston) were approached during their first trimester. Participants were eligible if they were at <15 weeks' gestation and had a history of GDM, family history of diabetes or GDM, or if they had BMI ≥25 kg/m2 and had one additional risk factor according to American Diabetes Association guidelines.<br>MOMS: Participants were eligible if they were receiving prenatal care at Massachusetts General Hospital. Participants provided written informed consent and were enrolled at their first prenatal visit where they donated an extra blood sample from a clinical blood draw. A subset of participants returned for a fasting blood draw at 16-20 weeks' gestation. Glucose and insulin were measured at that time in the hospital laboratory. For this study, we selected 55 participants who were subsequently diagnosed with GDM and had available stored samples and matched them in a 2:1 manner to participants who did not have GDM. We randomly selected controls from those with available samples based on our matching criteria but when there were multiple available matches we preferentially selected control participants who had both glucose and insulin levels available.
Ethics oversight	Gen3G: Each study participant provided informed written consent, and the study protocols were reviewed by the ethical committees from the CHUS, and from Harvard Pilgrim Health Care Institute.<br>SPRING and MOMS: approved by the Mass General Brigham Institutional Review Board (IRB)

Note that full information on the approval of the study protocol must also be provided in the manuscript.

Field-specific reporting

Please select the one below that is the best fit for your research. If you are not sure, read the appropriate sections before making your selection.

☒ Life sciences ☐ Behavioural & social sciences ☐ Ecological, evolutionary & environmental sciences

For a reference copy of the document with all sections, see nature.com/documents/nr-reporting-summary-flat.pdf

Life sciences study design

All studies must disclose on these points even when the disclosure is negative.

Sample size	Gen3G: for placenta RNAseq sample size = We included all samples that were collected, passed initial quality control RNA quality metrics in the lab (Bouchard), and pre-sequencing (Broad Institute), in addition to data quality control following RNA quantification. The final sample size of RNA sequencing data available was n=459, of which 434 participants had appropriate phenotype (Insulin sensitivity measure) for our main analyses of this study. For circulating IGFBP1 levels, we included all plasma samples available at first or second trimester for participants that we had second trimester oral glucose tolerance test information (for diagnosis of GDM); total sample size was n= 837 (Fig 2; Supp Table 3)<br>SPRING: We included 165 participants to measure circulating IGFBP1 based on available of plasma samples. Total SPRING enrollment was N=166, so there was only 1 participant without an available plasma sample.<br>MOMS: We included 55 participants who were subsequently diagnosed with GDM and had available stored samples and matched them in a 2:1 manner to participants who did not have GDM. We randomly selected controls from those with available samples based on our matching criteria but when there were multiple available matches we preferentially selected control participants who had both glucose and insulin levels available.

Data exclusions	Placenta RNA sequencing dataset: Following quantification, we applied additional quality control (QC) steps. Of the 466 samples sequenced, we excluded those with >1% of outlier genes (>3 times the inter quartile range (IQR) above Q3 or >3 IQR below Q1), leaving 459 samples for our final analytical data set. Among these, we had complete data on the phenotype of interest (Matsuda index) and covariates for 434 samples. Prior to differential gene expression analysis, we removed genes with low abundance, keeping only those genes with at least a count of 6 reads and a transcript per million (TPM) values >0.5 in a minimum of 20% of samples, as well as average mappability >0.8. After QC, 15,202 genes remained.
Replication	We measured circulating IGFBP1 in plasma samples of Gen3G participants to test associations with insulin sensitivity during prengancy and with risk of gestational diabetes.<br>We replicated our analyses and findings using two independent cohorts: SPRING and MOMS
Randomization	Our study is based on observational cohorts and did not include assessment of intervention(s), thus randomization is not relevant (not a clinical trial)
Blinding	Our study is based on observational cohorts and did not include assessment of intervention(s), thus blinding is not relevant

Reporting for specific materials, systems and methods

We require information from authors about some types of materials, experimental systems and methods used in many studies. Here, indicate whether each material, system or method listed is relevant to your study. If you are not sure if a list item applies to your research, read the appropriate section before selecting a response.

Materials & experimental systems

n/a	Involved in the study
☐	☒ Antibodies
☒	☐ Eukaryotic cell lines
☒	☐ Palaeontology and archaeology
☒	☐ Animals and other organisms
☒	☐ Clinical data
☒	☐ Dual use research of concern
☒	☐ Plants

Methods

n/a	Involved in the study
☒	☐ ChIP-seq
☒	☐ Flow cytometry
☒	☐ MRI-based neuroimaging

Antibodies

Antibodies used	We measured circulating IGFBP1 in plasma samples from all 3 cohorts in the same laboratory using a commercially available ELISA that measures free IGFBP1 (Catalog # DGB100, R&D systems, MN). The precision for the assays were: intra-assay CVs 5.6% and inter-assay CVs of 9.5%. We measured IGFBP1 levels in a blinded fashion, and we followed protocol for measurement per manufacturer's instructions.
Validation	n/a

Plants

Seed stocks	*Report on the source of all seed stocks or other plant material used. If applicable, state the seed stock centre and catalogue number. If plant specimens were collected from the field, describe the collection location, date and sampling procedures.*
Novel plant genotypes	*Describe the methods by which all novel plant genotypes were produced. This includes those generated by transgenic approaches, gene editing, chemical/radiation-based mutagenesis and hybridization. For transgenic lines, describe the transformation method, the number of independent lines analyzed and the generation upon which experiments were performed. For gene-edited lines, describe the editor used, the endogenous sequence targeted for editing, the targeting guide RNA sequence (if applicable) and how the editor was applied.*
Authentication	*Describe any authentication procedures for each seed stock used or novel genotype generated. Describe any experiments used to assess the effect of a mutation and, where applicable, how potential secondary effects (e.g. second site T-DNA insertions, mosiacism, off-target gene editing) were examined.*
